# Epidermal growth factor receptor inhibitor in advanced basal cell carcinoma

**DOI:** 10.1002/ccr3.4021

**Published:** 2021-03-24

**Authors:** Amir Amirabadi, Amirhosein Alami, Hamid Ahanchian, Nazila Ariaee, Nasrin Moazzen

**Affiliations:** ^1^ Department of Internal Medicine Azad University of Medical Sciences Mashhad Iran; ^2^ Department of Biology Azad University of Medical Sciences Mashhad Iran; ^3^ Clinical Research Development Unit of Akbar hospital Mashhad University of medical Sciences Mashhad Iran; ^4^ Allergy Research Center Mashhad University of Medical Sciences Mashhad Iran

**Keywords:** Basal cell carcinoma, cetuximab, epidermal growth factor receptor inhibitor, skin cancer

## Abstract

Cetuximab can be used for the treatment of advanced basal cell carcinoma, especially when the patient cannot tolerate routine chemotherapy. Future studies are needed to confirm it.

## Graphical abstract

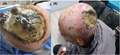

## 1 INTRODUCTION

Basal cell carcinoma (BCC) is a common cancer in human beings. Topical 5‐Fluorouracil (5‐FU) has been currently approved by the food and drug administration for the treatment of superficial BCCs. The most common systemic chemotherapy protocol is cisplatin‐based chemotherapy however, it is not used commonly. In a condition in which these drugs are contraindicated due to either a coexisting condition or a refractory disease, other systemic agents such as cetuximab can be an option. This epidermal growth factor receptor inhibitor has also been used in the treatment of cutaneous squamous cell carcinoma (SCC). There are a few case reports about cetuximab efficacy in BCC. In this article, we present three patients with BCC that had good outcomes being treated with cetuximab.

BCC is a slow‐growing malignancy of nonkeratinizing cells that arises from the basal cell layer of the skin. This condition is the most common cancer in human beings that seems to approximately affect one‐fourth of all humankind and includes about 75% of skin cancers diagnosed in the United States.^1^ BCCs rarely metastasizes but it is locally invasive and it can result in severe morbidity through local recurrence and destruction of underlying tissues. Approximately half of BCC cases appears on the nose and ear but it can affect any part of the skin including nonsun‐exposed areas such as penis, scrotum, vulva, and perianal area.^2^ Excisional surgery, Curettage and Desicction (C&D), and cryosurgery have been used to treat circumscribed, noninfiltrating BCCs. Moh's Micrographic Surgery (MMS) is the chosen treatment method for all recurrent and infiltrative BCCs, particularly if a tumor is located on the face.^3^ The reason that MMS has been chosen is that this method facilitates optimal margin control and conservation of normal tissue in the management of nonmelanoma skin cancers (NMSC), so it has become the standard treatment in a variety of skin cancer subtypes.^4^ When surgery is contraindicated, radiation therapy is an option for treating primary BCC. Radiation therapy may be indicated postoperatively if margins are ambiguous or involved and may also be considered when surgery could cause functional impairment or require a substantial reconstructive procedure, for example, in the cases that it has involved the eyelid, canthus, or nasal ala.^5^ 5‐Fluorouracil (5‐FU) is an antimetabolite that has been used topically since the 1960s as a treatment for actinic keratosis. This drug is a pyrimidine analog that acts selectively during the S phase to reduce the production of DNA and RNA in rapidly dividing cells. 5‐FU is currently approved by the FDA for the treatment of superficial BCCs. Although there are few published data on the 5‐year recurrence rates of 5‐FU in the treatment of superficial BCCs, it remains a standard topical treatment for actinic keratosis. The range of total clearance rates varies from roughly 10%‐98.6%. The most systemic chemotherapy protocol is cisplatin‐based chemotherapy although it is not common.^6^, ^7^ In very rare cases, some other systemic agents such as cetuximab (ERBITUX) are tried as a treatment for selected patients.^8^

The recent study conducted in the oncology department of the educational hospital, affiliated to Mashhad Azad Medical School (22 Bahman hospital), Mashhad, Iran during years 2019 to 2020. The procedures were followed by radiation oncologists in this center, the treatment results of three patients are presented. The work has been reported in line with the PROCESS 2018 criteria.^9^ The consent to publish their cases was written by the patients before.

In our center, patients with squamous cell carcinoma and basal cell carcinoma who have not responded to routine surgical and radiotherapy treatments for any reason, inevitably underwent systemic treatment. Meanwhile, most patients are treated with chemotherapy based on platinums, and few of others receive other treatments. The results of our treatment with cetuximab were interesting because the studies on it were very limited and sometimes contradictory, and it seems that it can be considered as an acceptable treatment option.

Comorbidities in our elderly patients were diabetes mellitus, renal failure, and Alzheimer. They have received at least one advanced surgery and radiation therapy before it. Their diseases were advance but unfortunately, they could not tolerate routine systemic treatment. Size of skin lesions was between 8 and 12 centimeter and after administration of cetuximab their size decreased as much as 2‐3 cm in one patient and almost resolved in two patients.

## 2 CASE PRESENTATION

### 2.1 Case 1

**FIGURE 1:**
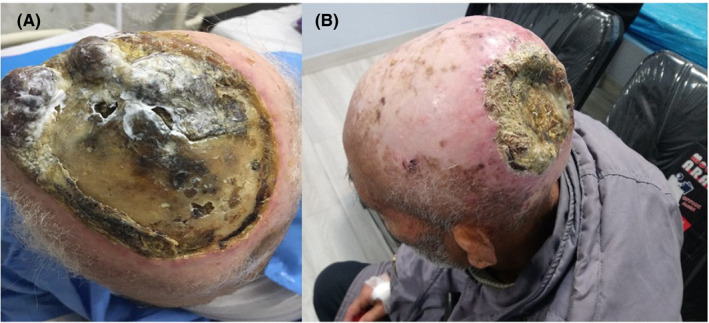
Case 1 on the right side (A), and case 2 in the left before treatment (B)

A 64 years old man was referred to our oncology clinic, with a large ulcerated lesion (12 cm in diameter) in his vertex. He had the lesion for 15 years. The patient had already received two cycles of superficial X‐ray radiotherapy in childhood due to Tinea Capitis infection. In the 50s and 60s, superficial X‐ray radiotherapy was considered as a standard treatment for fungal infections of the head that were resistant to conventional medications and was used in the treatment of this common infection till the evolution in the medical treatment of fungal infections in the mid‐20th century. Our patient suffered from a scaling patch in the vertex with pruritus and dryness sensation during his adulthood and later in life. The patch became ulcerative with an infected appearance in the last 15 years. He mentioned that the ulcer has got larger and he has been experiencing frequent bleeding episodes from the lesion. In the last 10 years, he received routine treatments for BCC of the skin including topical 5FU, three extensive surgeries with the use of skin flap in two out of three surgeries and two cycles of radiotherapy with linear accelerating (LINAC) device. The first cycle of radiotherapy was performed using 8 Mega electron volt (MeV) with a total dose of 50 Grays in 20 fractions, and the second cycle was performed after four years from the first cycle in 20 sessions using X‐ray 6 MeV with the final dose of 60 Grays. He also underwent 6 courses of Cisplatin‐based chemotherapy including Cisplatin 45 mg day (1‐3) and 5FU 900 mg day (1‐3) in the past year. Chemotherapy resulted in a slight initial response but had no long‐term response. The patient was referred to our oncology clinic for further treatment. We decided to administer cetuximab (700 mg loading dose based on body surface area calculation and weekly 430 mg). There was not any complication in treatment period within two months after initiation of chemotherapy, resolution of suppurative discharge and bleeding from the lesion was observed and after continuation of chemotherapy for 6 months, the inflammation and ulcer subsided and the primary lesion turned into a somewhat dry and almost asymptomatic patch. The patient was referred to a plastic surgeon to perform curative surgery for lesion excision and reconstruction of the lesion site. Figure 1 represents the case.

### 2.2 Case 2

A 59‐year‐old man was referred to our oncology clinic with a large ulcer (8 cm in diameter) in the right temporoparietal region. The lesion had massive supportive discharge in appearance. He also had received one course of superficial X‐ray radiotherapy in childhood due to Tinea Capitis infection. He informed us that the lesion became ulcerative 10 years ago. He was diagnosed to have type II diabetes mellitus at the age of 40 which was poorly controlled and resulted in diabetic nephropathy. He was under continuous dialysis three sessions per week based on conventional protocols for three years. A biopsy was obtained from the skin lesion which revealed BCC in histopathology. He underwent two surgeries 6 months and 2 years ago and 25 sessions of radiotherapy with LINAC with two beams of 6 mv X‐ray and a total dose of 62.5 Grays. Unfortunately, the lesion recurred after the primary remission despite these treatments; therefore, chemotherapy was recommended for the patient. Due to his renal insufficiency, chemotherapy regimens based on Cisplatin were not advisable; so, cetuximab (600 mg loading dose and 375 mg weekly doses) was initiated for the patient. There were grade 2 skin reactions in treatment course. Skin reactions due to cetuximab are acneiform lesions that diminished with local medication. In our patient, some days administration of topical Pliazon was effective. An acceptable response was observed for 6 weeks from the initiation of therapy. The patient is now under his 5th month of treatment and the lesion is almost dry and the ulcer subsided significantly without bleeding. Cetuximab will continue for one more month, and the patient will be referred to a plastic surgeon for curative surgery. Figure 1 represents the case.

### 2.3 Case 3

An 86‐year‐old man was referred to our oncology clinic with a large lesion in the forehead from 3 years ago. He also suffers from Alzheimer's disease and lives alone in his own house. He told us that he started to have a lesion 10 years ago on his forehead region and the recent extension of the lesion destroyed the right eyebrow and superior eyelid. The surgical consult revealed that he is nonoperable due to his compromised cardiorespiratory condition. The biopsy from the lesion revealed basal cell carcinoma. The patient refused to undergo radiotherapy, therefore, treatment with cetuximab (600 mg initial dose and 375 mg weekly doses) was chosen for him. Signs of lesion improvement were observed during the 6th and 8th weeks of treatment course. There were not any complications and he is now undergoing the 4th month of cetuximab. At present, the borders of the lesion are free from tumoral inflammation and the center of the lesion is heeling.

## 3 DISCUSSION

Biologic drugs are usually proteins or peptides with potential therapeutic effects induced by the interaction between known ligands or receptors. Cetuximab is an epidermal growth factor receptor (EGFR) inhibitor that is approved by the FDA for the treatment of metastatic colorectal cancer and head and neck cancer. This drug has also been used to treat cutaneous SCC. There are a few case reports on cetuximab efficacy in BCC. Because of the higher level of expression of the epidermal growth factor receptor in SCC and BCC, there is a hypothesis that inhibition of this receptor may have a therapeutic effect. In our patients, we have administered cetuximab for a couple of reasons, first of all, they had some underlying conditions that made it impossible for them to tolerate routine chemotherapy. Secondly, cancerous lesions were resistant to radiotherapy or patients refused to undergo radiotherapy. Therefore, we decided to administer cetuximab with a 400 mg per square meter loading dose and then 250 mg per square meter weekly. All three patients have had a very good response to this management and indeed tumor regression has occurred a few weeks after the treatment had initiated.

It seems that in advanced basal cell carcinoma of the scalp, in which the routine treatments such as radiotherapy and surgery have not been effective, using targeted therapy instead of systemic chemotherapy might be safer and have less complications. In the patients above, due to aging and underlying diseases, it was not possible to perform systemic platinum‐based chemotherapy. We hope that with the positive results we found in these three cases, along with other studies, may suggest a new option for the treatment of patients with advanced or resistant basal cell carcinoma to the cancer therapists all around the world.

## CONFLICT OF INTEREST

None to be declared.

## AUTHOR CONTRIBUTIONS

All the authors were involved in data recruiting. Amir Amirabadi, Nasrin Moazzen and Amirhosein Alami drafted the report and Hamid Ahanchian and Nazila Ariaee revised the munscript. All of the authors approved final paper.

## Data Availability

Data is available on Journal request.
